# Long noncoding RNA MALAT1 regulates HDAC4‐mediated proliferation and apoptosis via decoying of miR‐140‐5p in osteosarcoma cells

**DOI:** 10.1002/cam4.1677

**Published:** 2018-08-09

**Authors:** Yuxiu Sun, Baoli Qin

**Affiliations:** ^1^ Department of Internal Medicine Cancer Hospital of China Medical University Liaoning Cancer Hospital and Institute Shenyang China

**Keywords:** HDAC4, MALAT1, miR‐140‐5p, osteosarcoma, proliferation/apoptosis

## Abstract

Noncoding RNAs regulate the initiation and progression of osteosarcoma (OS). The role of long noncoding RNA metastasis‐associated lung adenocarcinoma transcript 1 (MALAT1) playing in OS and whether the function it working out was achieved through HDAC4 pathway remain uncovered. In this study, we illustrated that MALAT1 was upregulated and was correlated with poor prognosis in OS patients. Meanwhile, we demonstrated that a depression of MALAT1 suppressed proliferation and promoted apoptosis in OS cell line HOS and 143B. Further, we verified that MALAT1 exerting its function via upregulating of histone deacetylase 4 (HDAC4). Through an online prediction, a series of luciferase assays and RNA pull‐down assays, we demonstrated that both MALAT1 and HDAC4 were the targets of microRNA‐140‐5p (miR‐140‐5p) via sharing a similar microRNA responding elements. Even further, we revealed that MALAT1 served as a ceRNA of HDAC4 via decoying of miR‐140‐5p. Finally, we proved that MALAT1 promoted OS tumor growth in an in vivo animal study. In summary, the outcomes of this study demonstrated the complex ceRNA network among MALAT, miR‐140‐5p, and HDAC4‐mediated proliferation and apoptosis in OS. This study might provide a new axial in molecular treatment of OS.

## 1 INTRODUCTION

Osteosarcoma (OS) is the most prevalent primary sarcoma which produces bone or osteoid in young adolescence.^1^ For its highest aggressive biological behaviors, OS is always associated with an unfavorable prognosis and with a high rate of disability in youth.^2^ Although a combination of surgical resection and chemotherapy improve the survival rate of affected individuals, the average survival period of OS remains low.^3^, ^4^ Therefore, identification of new targets for molecular treatment of OS is urgently needed for both clinical doctors and involved patients.

Histone deacetylase 4 (HDAC4) plays global roles in the regulation of gene transcription, cell growth, survival and proliferation, and their aberrant expressions or activities contribute to cancer development.^5^, ^6^, ^7^, ^8^, ^9^, ^10^ Zeng LS revealed that upregulated HDAC4 is associated with higher tumor grade, advanced clinical stage, and poor survival rate in esophageal carcinoma, also they found that HDAC4 promoted proliferation and G1/S cell cycle progression in EC cells.^11^ Vallabhapurapu SD demonstrated that HDAC4 and its downstream complex maintained repressive chromatin around pro‐apoptotic genes Bim and BMF and regulates multiple myeloma (MM) survival and growth.^12^ Marroncelli N reported that HDAC4 regulated satellite cell proliferation and differentiation by targeting P21 and Sharp1 genes.^13^ Wilson AJ reported that HDAC4 promoted colon cancer cell proliferation via repression of p21.^14^ In a colorectal cancer study, Wei JY illustrated that nuclear localization of HDAC4 was required for melatonin‐induced apoptosis.^15^ To date, related researches on HDAC4 and OS cell proliferation and apoptosis are rare.

Long noncoding RNAs (lncRNAs) are a class of non‐protein‐coding transcriptions with the length of more than 200 nucleotides.^16^ It is widely reported that lncRNAs are involved in multiple malignant tumors including OS.^17^, ^18^, ^19^, ^20^, ^21^, ^22^ Metastasis‐associated lung adenocarcinoma transcript 1 (MALAT1) is located at chromosome 11q13.1 and is discovered as a new cancer‐associated lncRNAs in various cancers.^23^, ^24^, ^25^, ^26^ Wang Y found that MALAT1 promoted OS cell metastasis and proliferation via serving as a ceRNA of ROCK1 and ROCK2.^21^ Luo W illustrated that MALAT1 contributed to an increased expression of transforming growth factor alpha (TGFA) and facilitated OS cell growth through an inhibition of miR‐376a.^27^ By far, whether MALAT1 could regulate OS cell proliferation and apoptosis via HDAC4 pathway remains ambiguous.

In this study, we illustrated an elevated MALAT1 and its function in OS cell lines. Also, we demonstrated the complex relationship among MALAT1, HDAC4, and miR‐140‐5p. This study developed a new working pathway of MALAT1 and displayed a new molecular target axial for treatment of OS.

## 2 MATERIALS AND METHODS

### 2.1 Patients and tissue samples

Forty‐two cases of OS tissue and paired para‐tumor tissue specimens were collected during tumorectomy at Liaoning Cancer Hospital & Institute between April 2012 and October 2017. All 42 cases of patients had a clear histologic diagnosis of OS and were staged according to TNM classification of the International Union Against Cancer (UICC). Written informed consents were signed by the patients whose tissue specimens were involved in this research. The Institute Research Medical Ethics Committee of Liaoning Cancer Hospital & Institute granted approval for this study.

### 2.2 Cell culture

Human osteoblast cell line hFOB 1.19 was cultured in DMEM/F12 (Gibco, El Paso, TX, USA), while human OS cell lines MG‐63, U2OS, HOS, and 143B were cultured in Dulbecco's modified Eagle's medium (DMEM, Gibco), and all culture mediums were supplemented with 10% (v/v) fetal bovine serum (FBS, Sigma, St. Louis, MO, USA), 100 IU/mL penicillin (Baomanbio, Shanghai, China), and 100 mg/mL streptomycin (Baomanbio). MG‐63, U2OS, HOS, and 143B were incubated at 37°C, while hFOB1.19 was incubated at 34°C in a humidified atmosphere containing 5% CO_2_. The cultured cells were passaged when they grew to 80% confluent.

### 2.3 Oligonucleotide transfection

MALAT1 and HDAC4 silencing plasmids short interfering RNAs (siRNA) (si‐MALAT 01, si‐MALAT 02, and si‐HDAC4) as well as negative control siRNA (si‐control), MALAT1 overexpression plasmids (oe‐MALAT1) were chemically constructed and synthesized by Guangzhou RiboBio Co., Ltd. (RiboBio, Guangzhou, China). MiR‐140‐5p oligonucleotides miR‐140‐5p mimics and mimic control, miR‐140‐5p inhibitors, and inhibitor control were purchased from GenePharma (Shanghai, China). When OS cells were cultured to 70‐80% confluence, the aforementioned MALAT1 and HDAC4 intervention plasmids or oligonucleotides were transfected into the cultured OS cells using Lipofectamine 3000 (Invitrogen, Carlsbad, CA, USA) according to the manufacturer's instructions.

### 2.4 Reverse transcription and quantitative real‐time PCR

The procedure was carried out as previously described.^20^ Total RNAs were isolated using TRIzol reagent (Invitrogen). cDNA was synthesized using a Takara RNA PCR kit (Takara, Dalian, China) according to the manufacturer's protocol. PCR reactions containing SYBR Premix Ex Taq II (Takara) were followed according to the manufacturer's manual. The U6 small nuclear RNA and GAPDH were used as internal controls. Primer sequences were synthesized by Guangzhou RiboBio Co., Ltd. (RiboBio) as listed in Table 1.

**Table 1:** Primer sequences for qRT‐PCR

Name	Forward primer(5′‐>3′)	Reverse primer(5′‐>3′)
MALAT1	ATCTGCAAAACAAAAACCCCT	GTCTCCGAAGACACAGAGACCT
HDAC4	CACGAGCACATCAAGCAACAA	CAGTGGTTCAGATTCCGGTGG
miR‐140‐5p	CAGTGGTTTTACCCTATGGTAG	ACCATAGGGTAAAACCACTGTT
U6	GCTTCGGCAGCACATATACTAAAAT	CGCTTCACGAATTTGCGTGTCAT
GAPDH	GGAATCCACTGGCGTCTTCA	GGTTCACGCCCATCACAAAC

### 2.5 Western blot analysis

Total proteins were harvested using radio immunoprecipitation assay (RIPA) lysis buffer (Sigma) and qualified by a BCA detecting kit (Keygen, Nanjing, Jiangsu, China) according to the manufacturer's protocol. Protein samples were subjected to 10% SDS‐PAGE and transferred onto a PVDF (Amresco, Washington, DC, USA) membrane and then incubated with anti‐HDAC4 antibody (Abcam, Cambridge, MA, USA; dilution rates of 1:500) at 4°C overnight. The membranes were incubated with secondary antibodies (Abcam, dilution rates of 1:2000) at 25°C for 1 hour on the following day. Protein bands were detected on X‐ray film using an enhanced chemiluminescence detection system.

### 2.6 RNA in situ hybridization and immunohistochemistry analysis

The procedure was performed as previously described. Briefly, fresh OS tissue slices were washed with a solution of 0.5% Triton X‐100 in 1 × PBS and then incubated with anti‐MALAT1 probe (RiboBio) in a hybridization solution containing 1% blocking solution in a humid chamber at 37°C overnight. The following day, the slices were orderly washed with a solution of 0.1% Tween‐20 in 4 × sodium citrate buffer (SSC) for 5 minutes, a solution of 0.1% Tween‐20 in 2 × SSC for 5 minutes, and a solution of 0.1% Tween‐20 in 1 × SSC for 5 minutes at 42°C in dark. Lastly, the slices were triply rinsed with 1 × PBS for 5 minutes at room temperature and were counterstained by DAPI (Cell Signaling Technologies, Danvers, USA). All slices were observed and photographed under a microscope (Leica, Wetzlar, Germany).

The procedure of immunohistochemistry was performed as previously described.^28^

### 2.7 5‐ethynyl‐20‐deoxyuridine (EDU) incorporation assay

The procedure was carried out as previously described.^29^ HOS and 143B cells after different MALAT1, HDAC4, or miR‐140‐5p intervene were seeded in 96‐well plate and incubated for 48 hours. The following assays were performed according to the manufacturer's instructions by applying an EDU detection kit (Keygen). The nuclei were observed under a fluorescent microscope (Leica, Wetzlar, Germany). The quantitative data were expressed as the percentage of EDU‐positive nuclei relative to total number of nuclei counted.

### 2.8 Terminal deoxynucleotidyl transferase (TdT) dUTP Nick‐End Labeling (TUNEL) assay

The procedure was carried out as previously described.^30^ In briefly, HOS and 143B cells after different MALAT1, HDAC4, or miR‐140‐5p intervene were seeded on coverslips and incubated for 48 hours. All coverslips were fixed using of 4% paraformaldehyde for 30 minutes and then were permeabilized with 0.1% Triton X‐100 for 2 minutes on ice. Next, the cells were labeled using of a TUNEL kit (Keygen) according to the manufacturer's protocol. The apoptotic index was calculated using the following formula: apoptotic index = (total number of apoptotic cells/total number of cells) × 100% and then normalized to the control group.

### 2.9 Dual‐luciferase reporter assay

Wild and mutant reporter plasmids of MALAT1/HDAC4 wt‐MALAT1‐luc/wt‐HDAC4‐luc and mut‐MALAT1‐luc/mut‐HDAC4‐luc which containing a wild or a mutant miR‐140‐5p binding sites were synthesized by GenePharma (GenePharma, Shanghai, China). The procedure was carried out as previously described.^31^ In brief, when HOS and 143B cells grew to 70% confluence, wt‐MALAT1‐luc/wt‐HDAC4‐luc, and mut‐MALAT1‐luc/mut‐HDAC4‐luc were cotransfected with miR‐140‐5p mimics or mimic control using a Lipofectamine 3000 (Invitrogen), individually. Forty‐eight hours later, luminescence changes in each group were determined using a Dual‐Luciferase Reporter Assay System (Promega, Madison, WI, USA) according to the manufacturer's protocol.

### 2.10 RNA pull‐down assay

The procedure was carried out as previously described.^32^ Wt‐MALAT1 and mut‐MALAT1 as well as wt‐HDAC4 and mut‐HDAC4 were transcribed from vector pGEM^®^‐T (Promega) and biotin‐labeled with the Biotin RNA Labeling Mix (Roche, Basel, Switzerland) and T7 RNA polymerase (Roche), treated with RNase‐free DNase I (Roche), and purified with an RNeasy Mini Kit (Qiagen, Valencia, CA, USA). The biotinylated MALAT1 and HDAC4 probes were dissolved in binding and washing buffer, and incubated with Dynabeads M‐280 Streptavidin (Invitrogen) at 25°C for 10 minutes to generate probe‐coated beads according to the manufacturer's protocol. Then, HOS and 143B cell lysates were incubated with the probe‐coated beads, and the RNA complexes bound to these beads were eluted and extracted for qRT‐PCR analysis to detect the relative expression of miR‐140‐5p.

### 2.11 Xenograft nude mouse model

Female nude mice (4‐5 weeks old) were purchased from Animal Care and Use Committee of China Medical University. Ltd. and were fed under a condition of sterile specific pathogen‐free. About 1 × 10^6^ HOS cells with stable overexpression of MALAT1 and cells with blank vector in 50% Matrigel (BD Bioscience, New Jersey, USA) were into axilla of mice subcutaneously. The formatted tumors were harvested at day 7, 11, 15, and 19, respectively, for further detection. Tumor volume was measured using the formula of 1/2 (length × width^2^). All experimental procedures were carried out in compliance with the guiding principles for the Care and Use of Animals described in the American Journal of Physiology and with the Guidelines established by the Institute of Laboratory Animal Sciences, Faculty of Medicine, Kagoshima University. All efforts were made to minimize animal suffering, to reduce the number of animals used, and to utilize possible alternatives to in vivo techniques.

### 2.12 Statistical analysis

All experiments were repeated in triplicate, and all data from three independent experiments were expressed as mean ± SD. GraphPad Prism V5.0 (GraphPad Software, Inc., USA) software and SPSS 19.0 statistical software were used for statistical analysis. Pearson's chi‐squared test or Fisher's exact test were used to analyze the correlation between MALAT1 and clinicopathological features of OS patients, meanwhile, log‐rank test was used for survival analysis using of GraphPad Prism V5.0. Differences in two groups were analyzed by with the Student's t‐test or one‐way ANOVA. Differences were considered significant if *P* < 0.05.

## 3 RESULTS

### 3.1 MALAT1 was elevated and correlated with poor prognosis in OS patients

**Figure 1:**
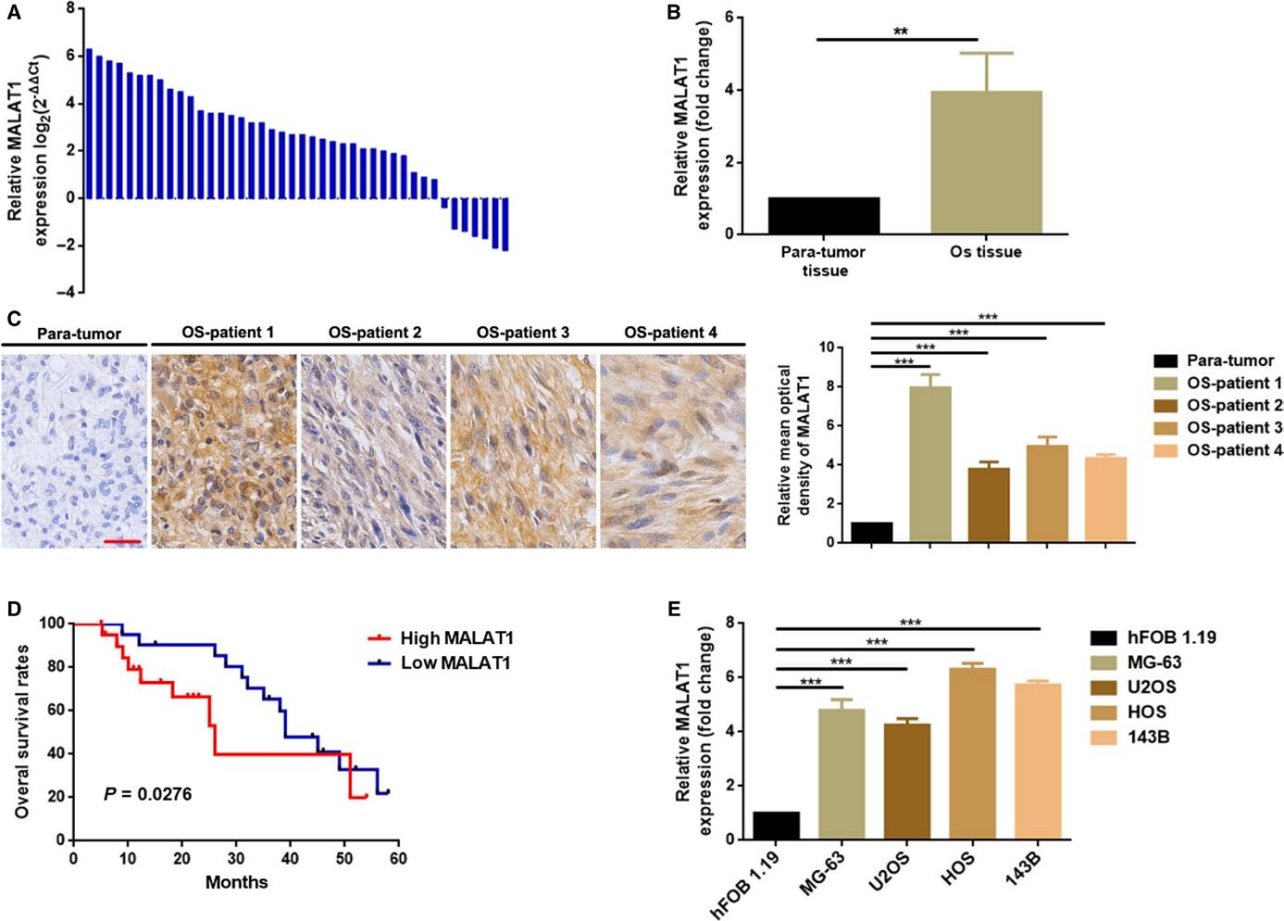
MALAT1 was elevated and correlated with poor prognosis in OS patients. A, B, Expression of MALAT1 in OS tissue specimens was determined by a qRT‐PCR assay, and data were shown as log_2_ (2^−△△Ct^) method (A) and △Ct method (B), respectively. **P <* *0.01 as normalizing and comparing to para‐tumor tissue group; C, MALAT1 was elevated in OS tissue specimens as measuring by an in situ hybridization assay. ***P <* *0.001 as normalizing and comparing to para‐tumor group; D, The overall survival (OS) of the patients with high MALAT1 was significantly shorter than that in the patients with low MALAT1, *P *=* *0.0276 as determined by Kaplan‐Meier analyses; E, MALAT1 expression was elevated in OS cell lines MG‐63, U2OS, HOS, and 143B. ***P <* *0.001 as normalizing and comparing to hFOB1.19 group. Data were shown as mean ± SD from three independent experiments

We first measured the expression level of MALAT1 in the collected 42 OS tissue specimens and paired para‐tumor tissue specimens by qRT‐PCR. As presented in Figure 1A,B, elevated MALAT1 was demonstrated in most OS tissue specimens (35/42, 83.33%). In addition, MALAT1 expression in tissue level was also determined by In situ hybridizations (ISH) analysis. As presented in Figure 1C, upregulated MALAT1 was shown in OS tissues as compared to para‐tumor tissues. Even further, we analyzed the correlation between MALAT1 expression and clinicopathological features of OS patients. As the data shown in Figure 1D and Table 1, higher MALAT1 was closely correlated with shorter survival rate (Figure 1D), advanced staging (IIB/III), bigger tumor size and distant metastasis (Table 2). Finally, we detected the expression of MALAT1 in 4 OS cell lines MG‐63, U2OS, HOS, and 143B and in a normal human osteoblastic cell line hFOB 1.19 by qRT‐PCR. As the results presented in Figure 1E, MALAT1 was significantly elevated in OS cell lines as compared to hFOB 1.19.

**Table 2:** Association of MALAT1 expression with clinicopathological features of osteosarcoma

Features	No. of cases	MALAT1		*P* value^a^
		High	Low	
Age at diagnosis				
<18	24	15	9	0.927
≥18	18	11	7	
Gender				
Female	22	14	8	0.808
Male	20	12	8	
Histological subtype				
Osteoblastic	8	5	3	0.765
Chondroblastic	10	5	5	
Fibroblastic	11	8	3	
Mixed	13	8	5	
Clinical stage				
I+IIA	23	18	5	0.016
IIB/III	19	8	11	
Distant metastasis				
Absent	22	17	5	0.031
Present	20	9	11	
Tumor size (cm)				
<5	25	19	6	0.023
≥5	17	7	10	
Anatomic location				
Tibia/femur	23	13	10	0.429
Elsewhere	19	13	6	

a *P*‐value obtained from Pearson's chi‐square test or Fisher's exact test.

### 3.2 Knockdown of MALAT1 inhibited proliferation but promoted apoptosis in HOS and 143B cells

**Figure 2:**
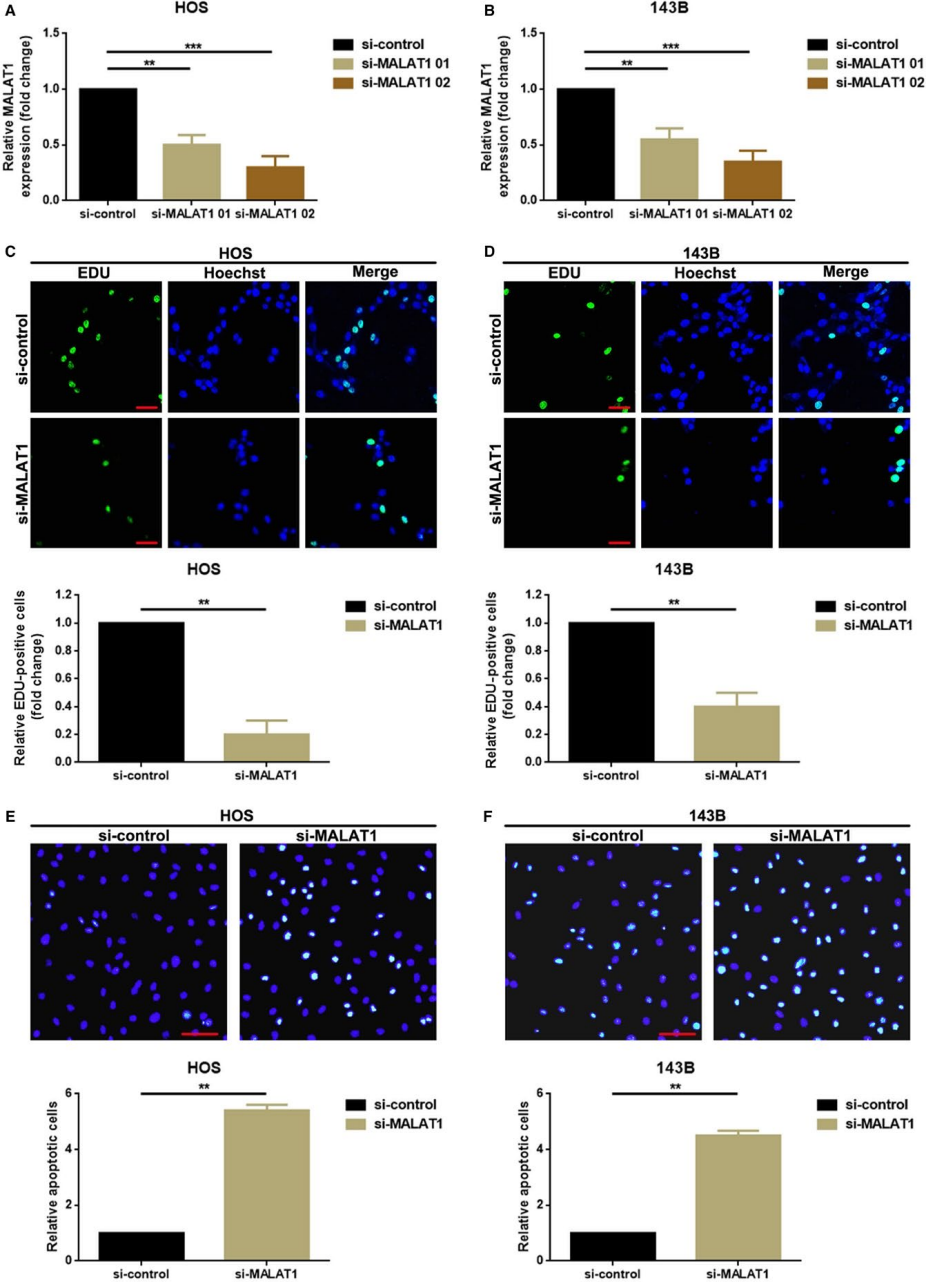
Knockdown of MALAT1 inhibited proliferation but promoted apoptosis in HOS and 143B cells. A, B, MALAT1 was knockdown by transfection of MALAT1 small interfering RNAs (si‐MALAT‐01 and si‐MALAT1‐02) which was confirmed by qRT‐PCR assays in HOS (A) and 143B (B) cells, respectively, and si‐MALAT1‐02 was selected in the following RNAi experiments for its higher silencing efficacy as comparing with si‐MALAT1‐01. **P <* *0.01 and ***P <* *0.001 as normalizing and comparing to si‐control group; C, D, Knockdown of MALAT1 remarkably inhibited HOS (C) and 143B (D) cells’ proliferation ability as determined by EDU assays; E, F, Knockdown of MALAT1 remarkably promoted apoptosis of HOS (E) and 143B (F) cells as determined by TUNEL assays. **P <* *0.01 as normalizing and comparing to si‐control group. Data were shown as mean ± SD from three independent experiments

In the up section, we elucidated a closely association between high MALAT1 and malignant features of OS especially in tumor size. Hence, we wondered the function of MALAT1 acting on OS cell proliferation and apoptosis. MALAT1 knockdown plasmids si‐MALAT1 were transected into HOS and 143B cells to downregulate the expression of MALAT1 in OS cells which were confirmed by qRT‐PCR (Figure 2A,B, as compared to si‐MALAT1 01, si‐MALAT1 02 presented a better silence efficiency and was chosen as the silencing tool in the following RNAi experiments). Further, EdU assays as well as TUNEL assays were applied to uncover the function of MALAT1 action on OS cell proliferation and apoptosis. As the outcomes presented in Figure 2C,D, knockdown of MALAT1 obviously inhibited OS cell proliferation capacity. Conversely, downregulation of MALAT1 promoted OS cell apoptosis which were elucidated by TUNEL assays (Figure 2E,F).

### 3.3 MALAT1 promoted proliferation but suppressed apoptosis via upregulating of HDAC4 in HOS and 143B cells

**Figure 3:**
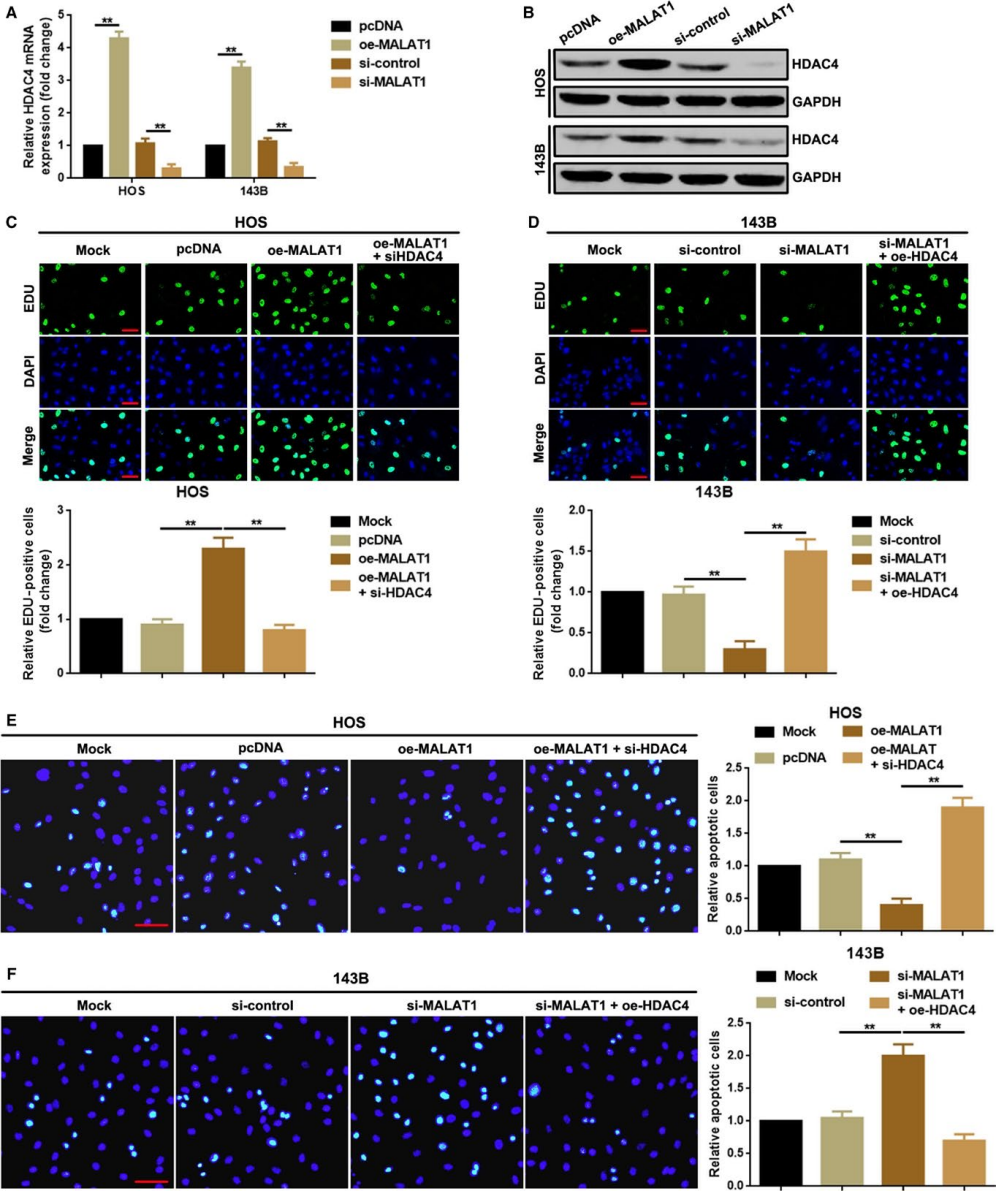
MALAT1 promoted proliferation but suppressed apoptosis via upregulating of HDAC4 in HOS and 143B cells. A, B, Overexpression and depression of MALAT1 positively regulated HDAC4 expression both in mRNA and in protein level as checked by a qRT‐PCR assay (A) and a Western blot (B). **P <* *0.01 as normalizing and comparing to pcDNA group; C, D, Overexpression of MALAT1 (oe‐MALAT1) promoted HOS (C) and 143B (D) cells’ proliferation but the facilitative effect was weakened by a knockdown of HDAC4 (oe‐MALAT1+ siHDAC4). **P <* *0.01 as normalizing to Mock group and comparing to oe‐MALAT1 group. (E, F) Overexpression of MALAT1 (oe‐MALAT1) inhibited HOS (C) and 143B (D) cells’ apoptosis but the suppressive effect was reversed by a knockdown of HDAC4 (oe‐MALAT1 + siHDAC4). **P <* *0.01 as normalizing to Mock group and comparing to oe‐MALAT1 group

According to previous researches, HDAC4, which was targeted by multiple miRNAs, was involved in proliferation and apoptosis in various malignant tumors including OS. In this section, we tried to elucidate whether the effect of MALAT1 acting on OS cell proliferation and apoptosis was achieved through HDAC4. We first demonstrated that upregulation of MALAT1 promoted HDAC4 expression and vice versa (Figure 3A,B). Further, we determined the character which HDAC4 might play in MALAT1‐associated proliferation and apoptosis. As the outcomes shown in Figure 3C, elevation of MALAT1 promoted HOS cell proliferation ability but the facilitative effect was attenuated by silencing of HDAC4 (cotransfection of oe‐MALAT1 and si‐HDAC4). On the contrary, downregulation of MALAT1 suppressed 143B cell proliferation but the suppressive effect was reversed by increasing of HDAC4 (cotransfection of si‐MALAT1 and oe‐HDAC4, Figure 3D). Even further, the apoptosis of HOS cells was inhibited by an upregulation of MALAT1 and this phenomenon was weakened by depression of HDAC4 (Figure 3E). Conversely, knockdown of MALAT1 boosted 143B cells apoptosis and an elevation of HDAC4 rollback the facilitative effect (Figure 3F). All the findings above indicated that MALAT1 exerted its function on proliferation and apoptosis via positive regulation of HDAC4.

### 3.4 MALAT1 and HDAC4 shared the same MREs for miR‐140‐5p

**Figure 4:**
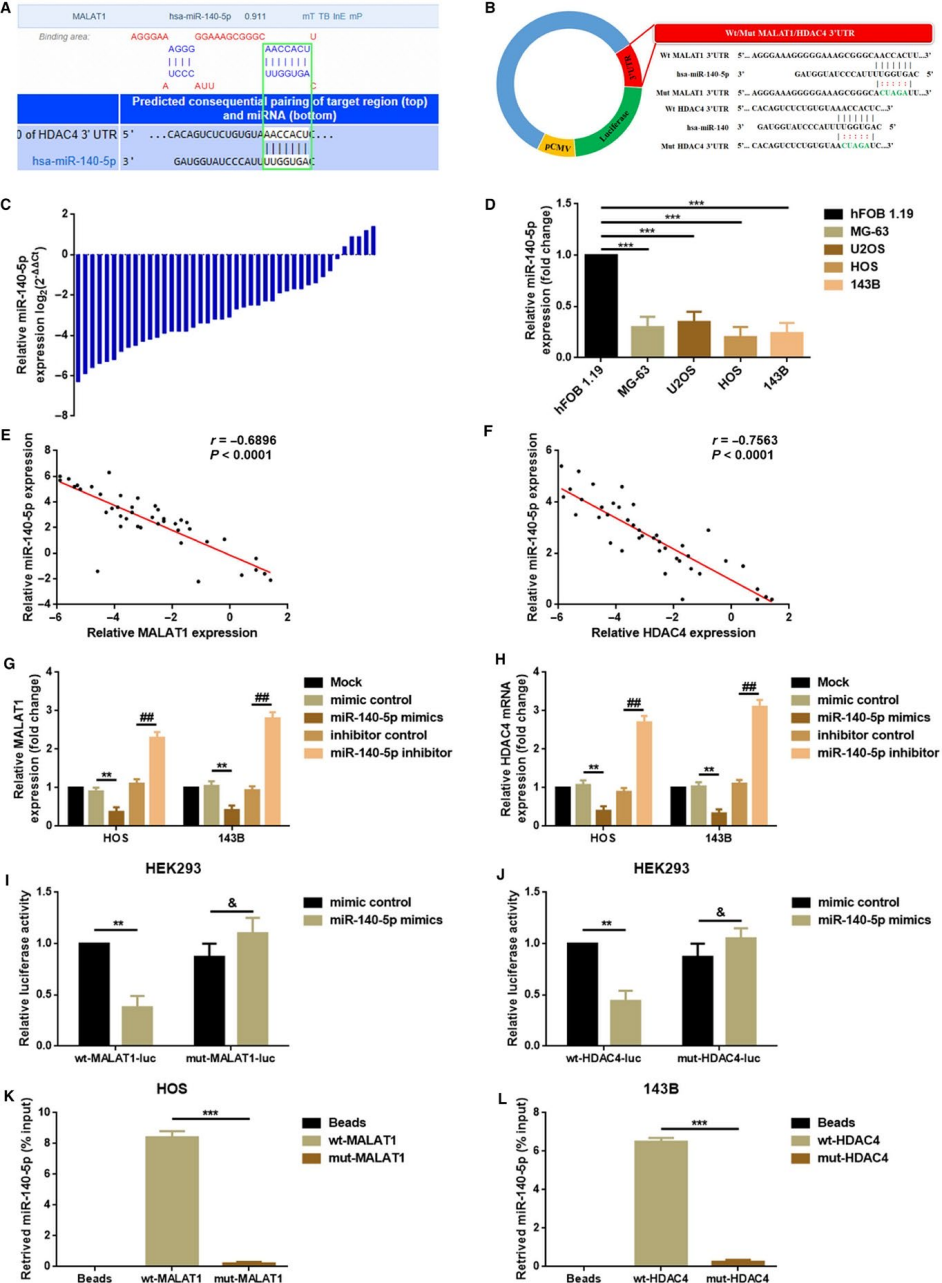
MALAT1 and HDAC4 shared the same MREs for miR‐140‐5p. A, HDAC4 and MALAT1 shared a similar miR‐140‐5p response elements (MRE‐140‐5p) as predicted by DIANA‐LncBase (http://carolina.imis.athena-innovation.gr) and Targetscan (http://www.targetscan.org/vert_71); B, Diagram of the wild and mutant luciferase reporter plasmids of MALAT1 and HDAC4; C, miR‐140‐5p was decreased in most OS tissue specimens (37/42, 88.10%) as determined by a qRT‐PCR assay, and data were shown as log_2_ (2^−△△Ct^) method; D, The expression of miR‐140‐5p was decreased in OS cell line MG‐63, U2OS, HOS, and 143B as detected by a qRT‐PCR assay. ***P <* *0.001 as normalizing and comparing to hFOB 1.19. E, F, The expression of MALAT1 and miR‐140‐5p as well as HDAC4 and miR‐140‐5p showed obviously negative correlations as determined by a Pearson's correlation analysis, *P *<* *0.0001; G, H, Up‐ and downregulation of miR‐140‐5p negatively affected MALAT1 (G) and HDAC4 (H) expression as measured by a qRT‐PCR assay. **P <* *0.01, ^##^ *P *<* *0.01 as normalizing to Mock group and comparing to mimic control and inhibitor control group, separately; I, J, Cotransfection of wt‐MALAT1‐luc and miR‐140‐5p mimics led to a remarkably weakening of luminescence and the phenomenon was dismissed when the theoretical binding sites for miR‐140‐5p in MALAT1 3′UTR were mutated. The same tendency was shown by a cotransfection of wt‐HDAC4‐luc/mut‐HDAC4 ‐luc and miR‐140‐5p. **P <* *0.01, ^&^ *P *>* *0.05 as normalizing and comparing to mimic control group; K, L, OS cell lysates were incubated with biotin‐labeled wt‐MALAT1/wt‐HDAC4 and mut‐MALAT1/mut‐HDAC4, respectively. Pulled‐down miR‐140‐5p was determined by a qRT‐PCR assay. The relative miR‐140‐5p which was pulled down by wt‐MALAT1 and wt‐HDAC4 was significantly higher than that was pulled down by mut‐MALAT1 and mut‐HDAC4. ***P <* *0.001 as normalizing and comparing to Beads group

It is widely known that lncRNAs could sponge certain microRNAs (miRNAs) and regulate their downstream genes via a mechanism of ceRNA.^32^ In a previous research, HDAC4 was reported as a target gene of miR‐140 and was involved in miR‐140‐mediated proliferation in OS cells.^33^ Through an online prediction, we first revealed that MALAT1 and HDAC4 shared similar theoretical microRNA response elements (MREs) for miR‐140‐5p in their 3′UTRs, respectively (Figure 4A). Second, we confirmed that miR‐140‐5p was decreased in OS tissue and cell lines (Figure 4C,D) and that there were negative correlations between miR‐140‐5p and MALAT1 or HDAC4 individually (Figure 4E,F). Third, we demonstrated that elevation and depression of miR‐140‐5p could negatively regulate MALAT1 and HDAC4 expression in mRNA level (Figure 4G,H). Lastly, RNA pull‐down assays and luciferase assays were performed to verify the targeted binding effect between miR‐140‐5p and 3′UTRs of MALAT1 or HDAC4. As the data presented in Figure 4I,J, cotransfection of wt‐MALAT1‐luc/wt‐HDAC4‐luc and miR‐140‐5p mimics led to a significant weakening of luminescence. When the theoretical miR‐140‐5p response elements in MALAT1/HDAC4 were mutated (cotransfection of mut‐MALAT1‐luc/mut‐HDAC4 ‐luc and miR‐140‐5p mimics), the luminescence was reinforced. Also, the outcomes of RNA pull‐down assays directly demonstrated that it was wt‐MALT1/wt‐HDAC4 but not mut‐MALAT1/mut‐HDAC4 could pull down miR‐140‐5p (Figure 4K,L). In brief, the outcomes of the present part indicated that MALAT1 and HDAC4 were both the targets of miR‐140‐5p via sharing a similar MRE.

### 3.5 MALAT1 regulated HDAC4 mediated proliferation and apoptosis via decoying of miR‐140‐5p

**Figure 5:**
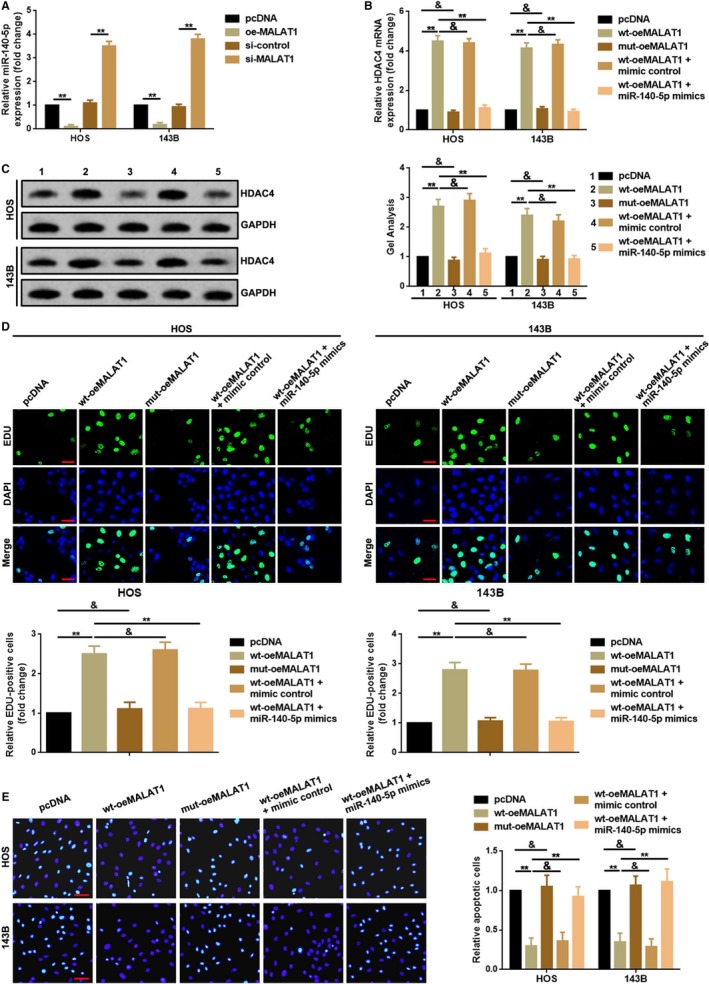
MALAT1 regulated HDAC4 mediated proliferation and apoptosis via decoying of miR‐140‐5p. A, Up‐ and downregulation of MALAT1 negatively affected miR‐140‐5p expression. **P <* *0.01 as normalizing and comparing to pcDNA group; B, C, Only wild type of MALAT1 overexpression plasmid (transfection of wt‐oeMALAT1) but not mut‐oeMALAT1 promoted HDAC4 expression, and the facilitative effect was attenuated by an upregulation of miR‐140‐5p (cotransfection of wt‐oeMALAT1 and miR‐140‐5p mimics) as detected by a qRT‐PCR assay (B) and a Western blot assay (C). **P <* *0.01, ^&^ *P *>* *0.05 as normalizing to pcDNA group; D, OS cells proliferation ability was promoted by a wild type (wt‐oeMALAT1) but not be done by a mutant type (mut‐oeMALAT1) of MALAT1 overexpression plasmids. And the facilitative effect wt‐oeMALAT1 did on OS cells’ proliferation was attenuated by an upregulation of miR‐140‐5p (cotransfection of wt‐oeMALAT1 and miR‐140‐5p mimics) which is determined by an EDU assay. **P <* *0.01, ^&^ *P *>* *0.05 as normalizing to pcDNA group; E, Only wt‐oeMALAT1 but not mut‐oeMALAT1 inhibited OS cells’ apoptosis, and the facilitative effect wt‐oeMALAT1 was reversed by an upregulation of miR‐140‐5p which was confirmed by a TUNEL assay. **P <* *0.01, ^&^ *P *>* *0.05 as normalizing to pcDNA group

In the previous sections, we proved that MALAT1 regulated HDAC4‐mediated proliferation and apoptosis and that MALAT1 was a target of miR‐140‐5p as HDAC4 did. We then attempted to figure out whether the role MALAT1 playing on HDAC4‐mediated proliferation and apoptosis was achieved by acting as a ceRNA of miR‐140‐5p. Primarily, we confirmed that up‐ and downregulation of MALAT1 also negatively affected the expression level of miR‐140‐5p (Figure 5A). Combined with the results of Figure 4G,H, we demonstrated the reciprocal inhibition effect between MALAT1 and miR‐140‐5p. Secondly, MALAT1 overexpression plasmids, wt‐oeMALAT1 and mut‐oeMALAT1 which containing wild and mutant miR‐140‐5p binding sites were constructed individually and an antisense experiment was executed to finally verify the network among MALAT1, miR‐140‐5p and HDAC4‐mediated proliferation and apoptosis. As shown in Figure 5B,C, it was wt‐oeMALAT1 but not mut‐oeMALAT1 that promoted HDAC4 expression both in mRNA and in protein level. More convincingly, the facilitative effect wt‐oeMALAT1 did on HDAC4 was reversed by an overexpression of miR‐140‐5p (cotransfection of wt‐oeMALAT1 and miR‐140‐5p mimics). Furthermore, we re‐executed EDU assay to validate the ceRNA nets (ceRNETs) among MALAT1, miR‐140‐5p, and HDAC4. As the outcomes demonstrated in Figure 5D, a transfection of wt‐oeMALAT1 significantly promoted OS cells’ proliferation ability, but a transfection of mut‐oeMALAT1 did not present the promoting effect. Meanwhile, the facilitative effect of wt‐oeMALAT1 working on proliferation was weakened remarkably by an upregulation of miR‐140‐5p. Finally, for apoptosis, the results of re‐performed TUNEL assay displayed a similar tendency as EDU assay did (Figure 5E).

### 3.6 MALAT1 promoted tumor growth in vivo

**Figure 6:**
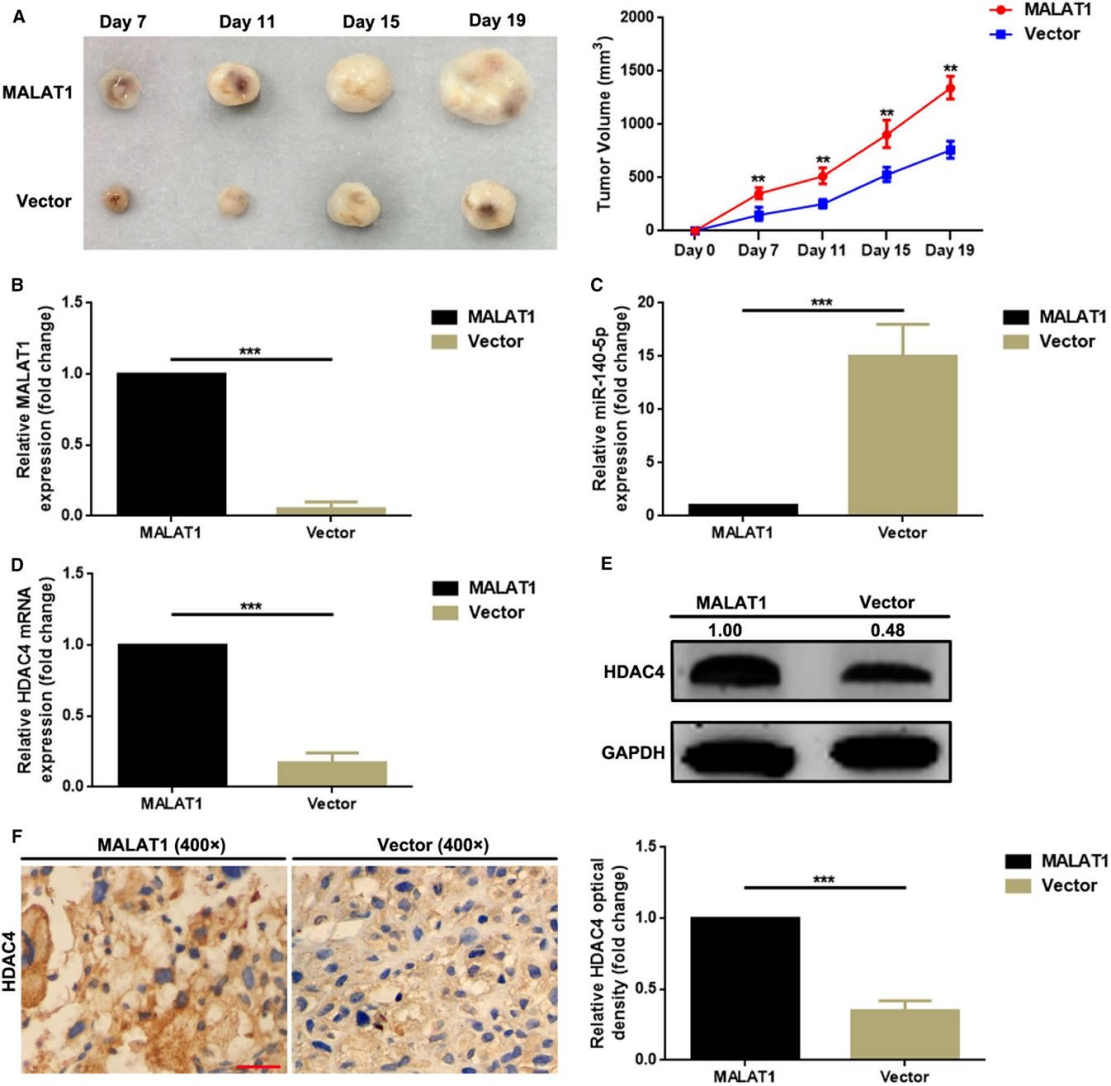
MALAT1 promoted tumor growth in vivo. A, Overexpression of MALAT1 obviously promoted OS tumor formation as presented by an in vivo animal study. (A—left, representative photographs of tumor formation in nude mice; A—right, growth curve of tumor volumes, **P <* *0.01 as comparing to vector group); B, C, Elevated MALAT1 (B) but depressed miR‐140‐5p (C) was found in the formatted tumor nodes as determined by a qRT‐PCR assay; D‐F, The expression of HDAC4 was also upregulated in the formatted tumor nodes as checked by a qRT‐PCR assay (D), a Western blot assay (E), and an IHC staining (F)

To finally confirm the facilitative role of MALAT1 working on OS in vivo, xenografts models in nude mice were applied. As the data presented in Figure 6A, overexpression of MALAT1 significantly promoted tumor growth. Meanwhile, the expression of MALAT1 and miR‐140‐5p in the formatted tumor nodes were measured by qRT‐PCR. As the outcomes displayed in Figure 6B,C, overexpression of MALAT1 remarkably inhibited miR‐140‐5p expression in vivo. Finally, HDAC4 in the formatted tumor nodes was determined. As shown in Figure 6D‐F, upregulation of MALAT1 promoted HDAC4 expression.

## 4 DISCUSSION

Accumulating evidence has shown that lncRNAs play vital roles in multiple biological processes including proliferation, apoptosis, angiogenesis, drug resistance, and metastasis in various malignant tumors.^34^, ^35^, ^36^, ^37^, ^38^ MALAT1 is a highly conserved lncRNA that was originally identified and is highly expressed in non‐small‐cell lung cancer.^39^ MALAT1 presented diverse regulative functions on multiple malignant tumors including OS.^40^, ^41^, ^42^, ^43^ In the present study, we also revealed the carcinogenetic effect MALAT1 working on OS especially on OS cell proliferation and apoptosis. Through a clinical case research and a series of in vitro cellular experiments, we found that MALAT1 was upregulated in OS and that an inhibition of MALAT1 suppressed proliferation but promoted apoptosis in OS cells. Meanwhile, we focused on the regulative effect of MALAT1 working on HDAC4—a proliferation and apoptosis‐related factor in manifold cancers. We showed that MALAT1 promoted HDAC4 expression and that an elevation of MALAT1 promoted proliferation but inhibited apoptosis in OS cells. Further, a knockdown of HDAC4 by RNAi attenuated the regulative effect which MALAT1 did on OS cell proliferation and apoptosis, and this phenomenon was a direct evidence that MALAT1 exerting its function through HDAC4 pathway.

The human HDAC4 gene, which is located on chromosome 2q37.3 and produced 8980 bp mRNA, spans approximately 353.49 kb and is involved in various cancers as being targeted by numerous miRNAs.^44^, ^45^, ^46^, ^47^ In the present study, we illustrated the negative correlation between HDAC4 and miR‐140‐5p. Through a luciferase assay and a RNA pull‐down assay, we confirmed that HDAC4 was a direct target of miR‐140‐5p. It is well accepted that an mRNA could be targeted by multiple miRNAs, and one miRNA could target different mRNAs. The reason why miR‐140‐5p was selected as a studying point in the present research was owe to the similar MREs it providing for both MALAT1 and HDAC4. As a member of miRNAs family, miR‐140‐5p is widely reported as a key regulator in many malignant tumors like non‐small‐cell lung cancer, gastric cancer, breast cancer, hypopharyngeal carcinoma and so on.^48^, ^49^, ^50^, ^51^ Fang Z reported that miR‐140‐5p suppressed gastric cancer's proliferation, migration, and invasion ability by targeting regulation of YES proto‐oncogene 1(YES1).^48^ In the present study, we illustrated that miR‐140‐5p was one of the “bridges” between MALAT1 and HDAC4.

**Figure 7:**
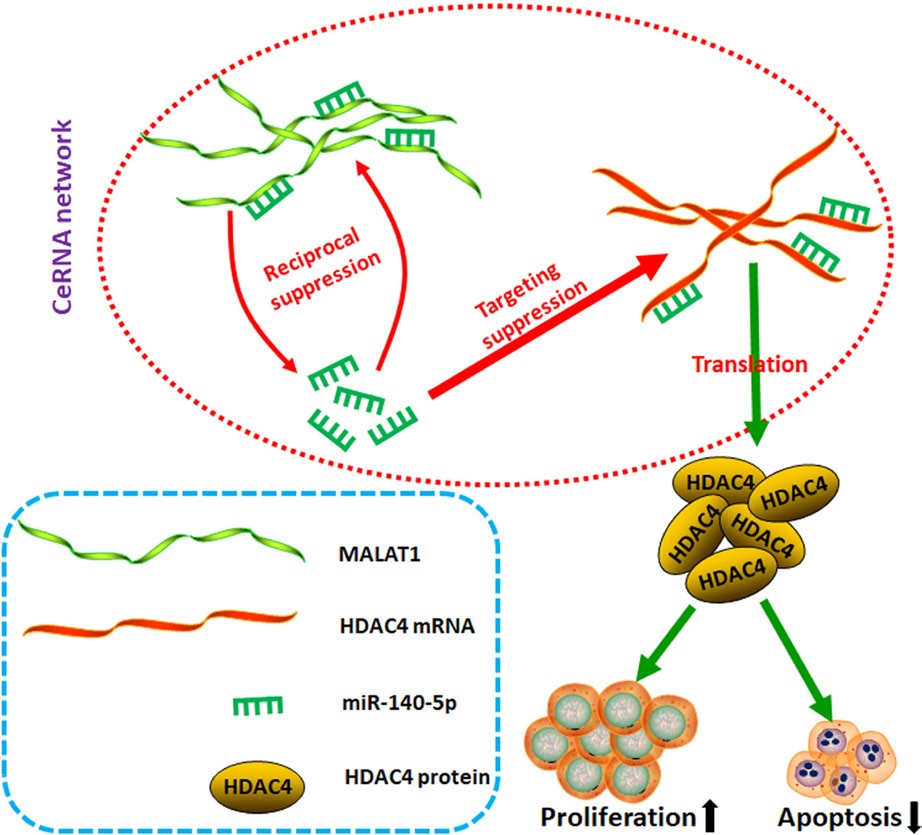
Schematic diagram of mechanism oh this research

As one of the most prevalent theory of how lncRNAs working out, competitive endogenous RNA (ceRNA) hypothesis was firstly proposed by Leonardo Salmena in 2011.^52^ CeRNA means all types of RNA transcripts could communicate with each other through a new “language” mediated by microRNA‐binding sites (“microRNA response elements,” or “MREs”). In the present study, we illustrated that MALAT1 could “talk” with miR‐140‐5p in a reciprocal inhibition manner. Further, we found that MALAT1 was a target of miR‐140‐5p. Through a series of antisense experiments, we displayed that an upregulation of miR‐140‐5p reversed the regulative effect of MALAT1 did on HDAC4‐mediated proliferation and apoptosis through acting with MALAT1 at miR‐140‐5p response element (MRE‐140‐5p). And these outcomes revealed the ceRNA network among MALAT, HDAC4, and miR‐140‐5p. Taking all, as the mechanism scheme shown in Figure 7, all the results of the present study indicated that MALAT1 regulated HDAC4‐mediated proliferation and apoptosis via decoying of miR‐140‐5p in OS cells.

Proliferation and apoptosis of OS are intricate biological processes which may involve in an army of molecules and factors. The present study just explored the function of MALAT1 did on OS proliferation/apoptosis and a downstream pathway of how it working. Our present research proposed a new targeted axial in treating of OS.

## CONFLICT OF INTEREST

The authors declare no conflict of interest.
